# From In Vitro Promise to In Vivo Reality: An Instructive Account of Infection Model Evaluation of Antimicrobial Peptides

**DOI:** 10.3390/ijms25189773

**Published:** 2024-09-10

**Authors:** Adam Carrera-Aubesart, Jiarui Li, Estefanía Contreras, Roberto Bello-Madruga, Marc Torrent, David Andreu

**Affiliations:** 1 Department of Medicine and Life Sciences, Universitat Pompeu Fabra, 08003 Barcelona, Spain; adam.carrera@upf.edu; 2 Department of Biochemistry and Molecular Biology, Facultat de Biociències, Universitat Autònoma de Barcelona, 08193 Bellaterra, Spain; jiarui.li@uab.cat (J.L.); roberto.bello@uab.cat (R.B.-M.); 3 Integrated Service for Laboratory Animals (SIAL), Faculty of Veterinary, Universitat Autònoma de Barcelona, 08193 Bellaterra, Spain; estefania.contreras@uab.cat

**Keywords:** AMPs, topoisomers, retroenantio, infection, murine model, crotalicidin

## Abstract

Antimicrobial peptides (AMPs) are regarded as a promising alternative to traditional antibiotics in the face of ever-increasing resistance. However, many AMPs fail to progress into clinics due to unexpected difficulties found in preclinical in vivo phases. Our research has focused on crotalicidin (Ctn), an AMP from snake venom, and a fragment thereof, Ctn[15-34], with improved in vitro antimicrobial and anticancer activities and remarkable serum stability. As the retroenantio versions of both AMPs maintained favorable profiles, in this work, we evaluate the in vivo efficacy of both the native-sequence AMPs and their retroenantio counterparts in a murine infection model with *Acinetobacter baumannii*. A significant reduction in bacterial levels is found in the mice treated with Ctn[15-34]. However, contrary to expectations, the retroenantio analogs either exhibit toxicity or lack efficacy when administered to mice. Our findings underscore the critical importance of in vivo infection model evaluation to fully calibrate the therapeutic potential of AMPs.

## 1. Introduction

Antimicrobial resistance (AMR), the ability of microorganisms to thrive in the face of antimicrobial agents [1], has grown into a severe global health threat. Projections suggest that by 2050 about 10 million lives might be endangered by AMR [2]. Agents for which AMR has been reported include practically all conventional antibiotics such as β-lactams [3], fluoroquinolones [4], rifamycins [5], macrolides [6], etc. Unfortunately, massive persistent misuse of antibiotics in both human and veterinary medicine keeps fueling AMR, a vicious circle that seems hard to interrupt unless innovative anti-infective therapies are soon brought into play.

In such a disquieting context, antimicrobial peptides (AMPs) have been heralded as promising candidates due to their efficacy, broad spectrum, and, especially, their mechanism of action-related low propensity to elicit resistance compared to conventional antibiotics [7]. These favorable outlooks have not yet translated into a plentiful AMP pipeline; to date, only seven AMPs have received FDA approval, namely gramicidin D [8], daptomycin [9], colistin [10], vancomycin [11], oritavancin, dalbavancin, and telavancin [12], with a few others such as plectasin [13], Dermegen^®^ [14] or MBI-226^®^ [15] in various phases of clinical trials.

AMPs are medium-size sequences (15–30 residues on average) typically rich in positively charged [16] as well as hydrophobic residues [17]. These compositions tend to favor AMPs to adopt amphipathic structures which play a crucial role in bacterial death [18,19], such as α-helical [20], β-sheet [21], mixtures of both [22], in either linear [23] or cyclic [24] arrangements.

**Figure 1:**
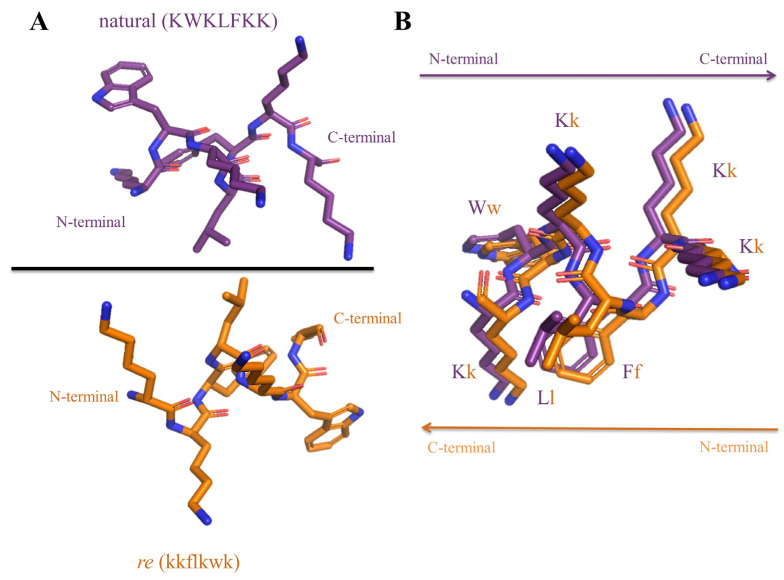
(**A**) *re* (kkflkwk) modification of the first part of the hybrid peptide CA(1-7)M(2-9) (KWKLFKK) [31] obtained using Pymol [32]. (**B**) Overlaying the canonical (purple backbone, conventional left-to-right orientation, and N- to C-terminus layout) with the *re* (orange backbone, right-to-left orientation, N- to C-terminus layout) version shows that the side chains adopt similar orientations while the amide bonds are reversed.

Part of the difficulties faced by AMPs aiming to reach the drug market have to do with their limited stability due to easy degradation by proteases in biological fluids [25]. This drawback, inherent to the AMP composition of L-amino acids, can be at times successfully addressed by incorporating D- instead of protease-sensitive L-residues at selected locations [26]. In fact, it is well established that enantiomer (all-D) forms of many AMPs are equipotent with the natural ones [27]. The D-enantiomers are one case of topoisomerism, a term that refers to three-dimensionally distinct versions of a molecule with the same composition. In addition to the enantio (*e*) form, the retro (*r*, reverse sequence, L-amino acids) and the retroenantio (*re*, reverse sequence, D-amino. acids) forms also fit the topoisomer description. Although the structural changes of a given peptide topoisomer are normally bound to translate into different biological effects, in the *re* form, a certain measure of topological similarity exists with the canonic form [28,29]: the side chains in both structures adopt superimposable orientations when the latter is plotted in a horizontally flipped fashion (Figure 1). Based on this spatial mimicry, various research efforts in recent years have sought to develop *re* peptides with improved activity [29,30].

In this work, we focus on crotalicidin (Ctn), a 34-residue AMP from the venom of *Crotalus durissus terrificus*, a South American pit viper [33,34]. Ctn exhibits potent antimicrobial activity against clinical or laboratory strains, especially Gram-negative ones. It is, however, slightly toxic to eukaryotic cells, including cancer cells [33,35], which makes it a good anticancer peptide (ACP) candidate. Virtual Ctn dissection with elastase yields two fragments, Ctn[1-14] and Ctn[15-34]. While the former (KRFKKFFKKVKKSV) retains the α-helical conformation of Ctn, it loses both the anticancer and antimicrobial properties. In contrast, less structured Ctn[15-34] (KKRLKKIFKKPMVIGVTIPF) remains comparable to Ctn on both antimicrobial and anticancer terms and exhibits minimal toxicity towards healthy eukaryotic cells. Notably, the Ctn[15-34] half-life in human serum is an unusual 770 min, compared with 71 min for full-sequence Ctn [33,36]. Mechanistically, both Ctn and its fragment Ctn[15-34] operate through a three-step pattern [37]: (1) initial peptide recruitment to the bacterial membrane by electrostatic interactions, (2) accumulation on the membrane surface, and (3) membrane disruption and ensuing bacterial cell death. In light of these findings, we explored the topoisomer strategy, specifically the *re* approach, on both peptides, testing them as AMPs and ACPs with promising outcomes. Furthermore, a remarkable property of Ctn[15-34] is its high stability in human fluids (Table 1) as reported earlier [33,36]. This level of proteolytic resistance is quite unusual among AMPs, which typically have much shorter half-lives (e.g., 60 min for LL-37 vs. 770 min for Ctn[15-34]). Additional stability studies [38] have likewise shown increased half-lives (>24 h) for the *re* versions. The next logical step after these results is the current work, where we explore the in vivo potential of Ctn and Ctn[15-34] and their *re* counterparts in a murine infection model with *Acinetobacter baumannii*, a pathogen in the ESKAPE group [39] and a top concern for the World Health Organization (WHO).

## 2. Results and Discussion

### 2.1. Peptides

Given the promising results of the *re* approach in previous studies [40,41,42], we decided to perform a murine infection study of Ctn and Ctn[15-34], in both native and *re* versions. From the eight peptides used in a previous study [38], the last three in Table 1 were selected for in vivo experiments based on MIC, cytotoxicity, and protease stability considerations. The original Ctn was not included due to its low stability in human serum.

### 2.2. Treatment with Ctn[15-34] Reduces Bacterial Levels in Females, Whereas Ctn re Does Not Improve the Survival Rate

A preliminary sighting study was conducted using the three peptides to establish an initial estimate of their maximum tolerance doses based on the in vitro findings from our previous study [38]. This assessment involved i.p. injections, with the initial dosages set at 30 mg/kg for Ctn[15-34] and Ctn[15-34] *re*, while Ctn *re* started at 3 mg/kg. This dosage was determined by the cytotoxicity assays in vitro (Table 1); accordingly, for Ctn[15-34] and Ctn[15-34] *re*, with the higher IC_50_, the initial dose was 30 mg/kg. For Ctn *re*, with an IC_50_ 10× lower than Ctn[15-34] *re*, the initial dose was 3 mg/kg.

In the experiment involving Ctn[15-34] *re*, it became evident that the peptide was toxic: an animal died within 3 min at 30 mg/kg, within 6 min at 15 mg/kg, and after 33 min at 5 mg/kg. Consequently, Ctn[15-34] *re* was excluded from further in vivo studies, while Ctn[15-34] and Ctn *re* were viable at the predicted dosages.

**Figure 2:**
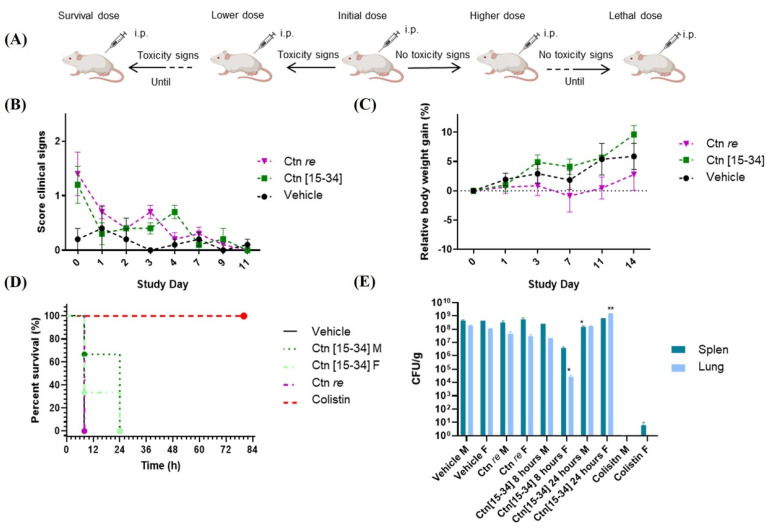
Results of the first in vivo study. (**A**) Scheme of the sighting study for toxicology assessment. (**B**) Average clinical scores and (**C**) relative BW gain (%) during the 14-day toxicology study. (**D**) Survival curve of infected mice treated with 30 mg/kg of Ctn[15-34], 3 mg/kg of Ctn *re*, 10 mg/kg of colistin or HBS vehicle. All animals were inoculated with 10^8^ CFU/kg *A. baumannii* with 5% mucin 2 h before treatment. Surviving animals were monitored for 3 days. (**E**) Average CFU/g in lung and spleen after efficacy study 1 endpoint. Significance estimated in comparison to vehicle (** *p* < 0.01, * *p* < 0.05).

In the tolerance assay 1, the results from the sighting study were confirmed. Animals received single doses of 30 mg/kg for Ctn[15-34] and 3 mg/kg for Ctn *re*. While exhibiting early clinical signs such as reduced motor activity, piloerection, and weight loss (see Figure 2B), most animals showed improved clinical conditions in the following days, including similar body weight (BW) evolution for Ctn[15-34] and Ctn *re* (Figure 2C).

The therapeutic effectiveness of Ctn[15-34] and Ctn *re* was evaluated in efficacy assay 1 using *A. baumannii* as a pathogen, with the 10^8^ CFU/kg inoculum determined by Li et al. [43], supplemented with 5% porcine mucin to induce immunosuppression, and an i.p. peptide dose of 30 mg/kg for Ctn[15-34] and 3 mg/kg for Ctn *re*. In the negative control group treated with HBS (vehicle), all animals exhibited severe clinical symptoms after 8 h, while in the positive control group (colistin), a 100% survival rate was observed. However, in the colistin-treated animals, an incidence of weight loss on the first day with subsequent recovery was found. There was also a slight initial increase in clinical scores, but the animals showed improvement later on.

Animals given Ctn *re* behaved similarly to the vehicle-treated group, being euthanized after 8 h due to clinical symptoms. In contrast, Ctn[15-34] showed a higher survival rate that allowed us to extend observation for 24 h (Figure 2D). As shown in Figure 2D,E, segregating into female (F) and male (M) categories allowed us to observe some differences in toxicity and survival outcomes.

After both efficacy studies on *A. baumannii*, left lung and spleen specimens were taken to determine infection levels by CFU (colony-forming units) counts. CFU/g values were in the 10^8^–10^9^ and 10^1^–10^2^ range, respectively, for the vehicle and the positive control (colistin) groups. Ctn *re* scored similarly to the vehicle-treated group, and so did Ctn[15-34] on male animals, but female mice showed a reduced bacterial count during an initial 8 h period, followed by a rise at 24 h (Figure 2E).

### 2.3. Treatment with Ctn[15-34] Thrice a Day Lowers Bacterial Load but Does Not Improve Survival Rate

**Figure 3:**
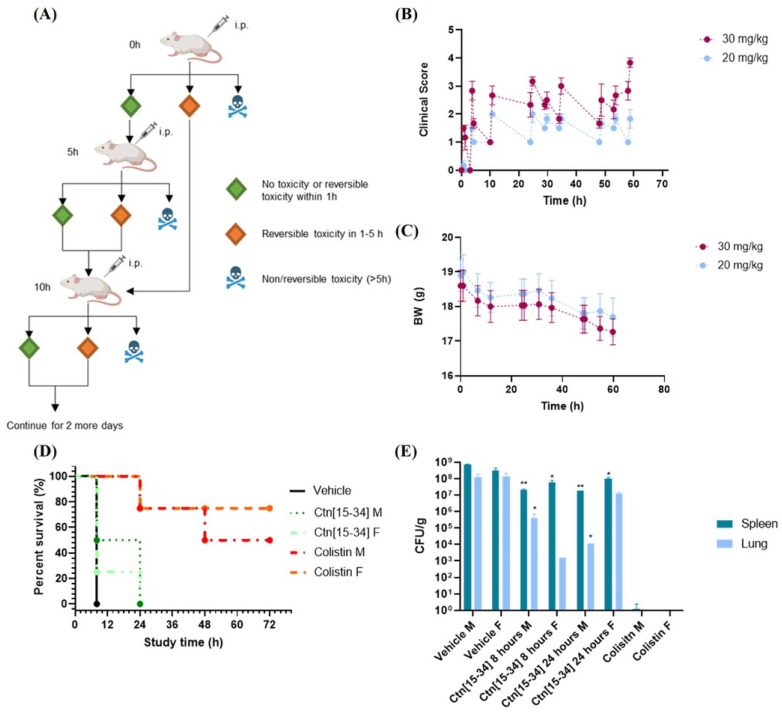
Results of the second in vivo study. (**A**) Scheme of the second toxicity assay. (**B**) Average clinical scores and (**C**) relative BW gain (g) for 30 mg/kg and 20 mg/kg thrice a day during the 3-day study. (**D**) Survival curve of infected mice treated with 20 mg/kg of Ctn[15-34], 10 mg/kg of colistin or HBS vehicle. All animals were inoculated with 10^8^ CFU/kg *A. baumannii* with 5% mucin 2 h before treatment. Surviving animals were monitored for 3 days. (**E**) Average CFU/g in lung and spleen after efficacy study 2 endpoint. Significance estimated compared to vehicle (** *p* < 0.01, * *p* < 0.05).

A second trial (efficacy study 2) was performed after a new toxicological assay for Ctn[15-34] only (tolerance assay 2), where peptide was given thrice daily for three consecutive days, at 30 and 20 mg/kg. In both dosage groups, a 6–9% BW loss during the first 24 h (Figure 3C) was observed, along with some toxicity signs (abdominal distension, resolving rapidly within 30 min; mucous feces, piloerection, abnormal behavior, and hunched posture). For the second group, these clinical signs were reversible and consistently lower than for the 30 mg/kg group (Figure 3B); hence, the lower dosage (20 mg/kg thrice daily for 3 days) was chosen.

In this study, animals given HBS had to be euthanized after 8 h due to the onset of clinical signs. Increased toxicity was also found with the thrice daily dose of colistin compared to the previous study, with severe clinical signs causing the termination of one male and one female animal at 24 h, and only 50% male and 75% female animals surviving at the endpoint (Figure 3D).

Ctn[15-34], for its part, performed comparably to the previous study, with some difference in survival rates between male (all euthanized after 8 h) and female animals (50% survival at 8 h, all euthanized by 24 h, Figure 3D).

The CFU/g counts in the lung and the spleen after the endpoint (Figure 3E) show a clear distinction between male and female animals treated with Ctn[15-34]. Although both groups had died by 24 h, bacterial counts at 8 h were lower in females than in males. Intriguingly, after 24 h, this difference was reversed. It is also worth noting that, in animals not surviving the thrice daily colistin dosage, bacterial presence in the spleen or lungs was nearly negligible, suggesting death from causes other than the infection, most likely nephrotoxicity and neurotoxicity [44,45].

### 2.4. Ctn[15-34] Reduces Levels of TNF-α in Mice after 8 h and Ctn[15-34] re Increase TNF-α Levels in LPS-Stimulated Human MNC Cells

**Figure 4:**
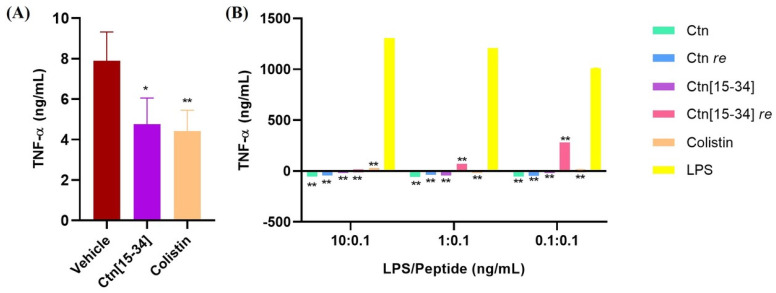
Evaluation of TNF-α levels. (**A**) Average TNF-α (ng/mL) concentration of mouse serum treated with vehicle, Ctn[15-34] and colistin; (**B**) TNF-α levels of LPS-stimulated human MNC in the presence of Ctn, Ctn *re*, Ctn[15-34], Ctn[15-34] *re*, and colistin. Significance estimated compared (**A**) to vehicle and (**B**) to LPS (** *p* < 0.01, * *p* < 0.05).

Blood samples were collected from each sacrificed animal in efficacy study 2 to measure TNF-α levels. The excessive production of pro-inflammatory cytokines, including TNF-α, is associated with septic shock, a critical factor contributing to mortality in Gram-negative infections [43]. As depicted in Figure 4A, Ctn[15-34] effectively reduces TNF-α levels in mice to a degree comparable to colistin.

The anti-endotoxin activity of the four peptides (Table 1) and colistin as the positive control was also assessed by measuring TNF-α levels in human MNC stimulated with different ratios of peptide/LPS. The results showed all peptides effectively reduced TNF-α levels compared to LPS alone. For Ctn[15-34] *re* (Figure 4B), particularly at LPS/peptide ratios of 0.1:0.1, TNF-α levels evidencing high inflammation and eventually leading to septic shock were found, compatible with the spontaneous deaths observed with this peptide.

### 2.5. Organ Staining Consistent with Bacterial Levels at Each Treatment

**Figure 5:**
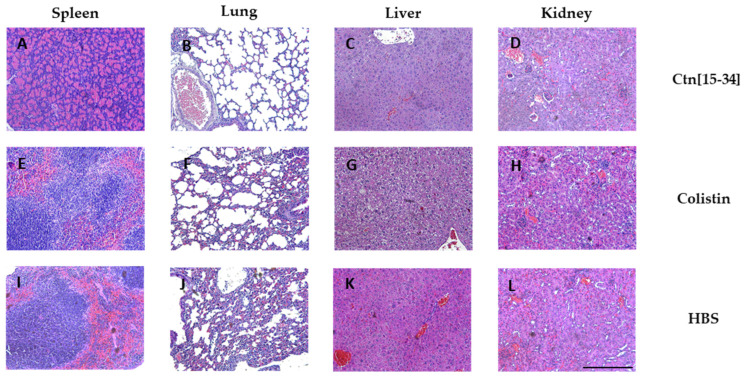
Hematoxylin and eosin-stained micrographs of spleen (**A**,**E**,**I**), lung (**B**,**D**,**J**), liver (**C**,**E**,**K**), and kidney (**D**,**H**,**L**) specimens. Scale bar 100 μm.

Hematoxylin and eosin staining of spleen, lung, liver, and kidney specimens was performed (Figure 5). While no lesions compatible with the experimental infection were observed, apoptosis was noted in the spleen lymphocytes of one animal each in the HBS (vehicle) and the Ctn[15-34] group.

**Figure 6:**
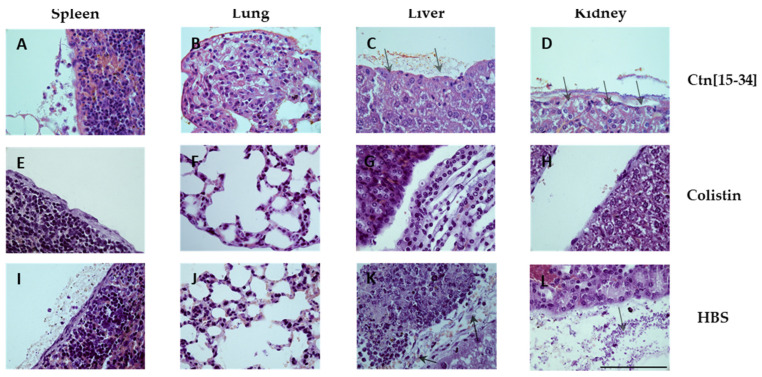
Gram-stained micrographs of spleen (**A**,**E**,**I**), lung (**B**,**D**,**J**), liver (**C**,**E**,**K**), and kidney (**D**,**H**,**L**) specimens. Scale bar 100 µm.

A Gram stain was also conducted (Figure 6) to assess the bacterial load in each organ. The organs of untreated mice exhibited bacterial accumulation (Figure 6I–L), indicating the spread of infection to other organs. In contrast, organs treated with Ctn[15-34] (Figure 6A–D) showed reduced bacterial accumulation, primarily in the tissue wall, suggesting partial efficacy though not complete elimination. As expected, the organs of colistin-treated mice showed no evidence of bacterial survival (Figure 6E–H).

## 3. Materials and Methods

### 3.1. Mice, Bacterial Strains and Other Material

Balb/c mice (9–12 weeks old, 15–28 g, male and female) were supplied by Charles River Laboratory; the study procedures were approved by the Animal and Human Experimentation Ethics Committee at Universitat Autònoma de Barcelona (UAB, Barcelona, Spain). The animals were acclimated at least 5 days from arrival until the start of the experiment with a free diet and water provided.

*A. baumannii* strains (CECT 452, Valencia, Spain, ATCC 15308, Manassas, VA, USA) were from the Colección Española de Cultivos Tipo (CECT, Valencia, Spain). Porcine mucin was from Sigma (St. Louis, MO, USA); Mueller-Hinton broth (MHB) from Merck (Darmstadt, Germany); agar from BD (Germiston South, South Africa); lysogeny broth (LB) from Nzytech (Lisbon, Portugal); and Hank’s balanced salt solution (HBS) from Cytiva (Fålhagen, Sweden).

The ELISA kit for TNF-α in human cells was Human TNF ELISA Kit II from BD Bioscience (Franklin Lakes, NJ, USA), and that for TNF-α in mouse blood was Mouse TNF Alpha ELISA Development Kit (ABTS) from LSBio (Lynnwood, WA, USA). ABTS Liquid substrate solution, BSA, Tween-20, and ELISA plates (439454) were from Sigma (St. Louis, MO, USA); Dulbecco’s PBS [×10] from Thermo Fisher (Waltham, MA, USA); and *E. coli* lipopolysaccharide (LPS) O111:B4 (O-LPS) was from Hycult Biotech (Uden, The Netherlands).

### 3.2. Peptides

Ctn, Ctn *re*, Ctn[15-34] and Ctn[15-34] *re* (sequences shown in Table 1) were produced by Fmoc solid phase synthesis as previously described [38]. The purified end products were satisfactorily characterized by HPLC and mass spectrometry (Table S2).

### 3.3. Minimum Inhibitory Concentration (MIC)

MIC was determined as described [17]. Peptide stocks in water were serially diluted from 100 to 0.2 μM and added to polypropylene 96-well plates (Greiner, Frickenhausen, Germany). Fresh bacteria were incubated at 37 °C in MHB up to exponential growth and diluted to a final inoculum of 5 × 10^5^ CFU/mL. BSA and acetic acid at 0.04% (*w*/*v*) and 0.002% (*v*/*v*) final concentration, respectively, were added to avoid peptide self-aggregation. Plates were incubated for 20–22 h at 37 °C. Each peptide was tested in duplicate. MIC values were defined as the lowest peptide concentration where bacterial growth was not detected.

### 3.4. Peptide Cytotoxicity

About 60,000 1BR3G human fibroblast cells were added to different microfuge tubes containing 2-fold serial dilutions of the peptide, with a final concentration in the range between 0.1 and 100 µM in RPMI containing 2% of FBS. After 30 min incubation at 37 °C and 5% CO_2_, 100 mL of medium containing approximately 10,000 treated cells were transferred to a 96-well plate. Then, 15 µL of Cell Titer Blue dye (Promega, Madison, WI, USA) was added, and plates were reincubated for 24 h. Fluorescence was read at 4 and 24 h after dye addition in a Synergy HTX (BioTek, Winooski, VT, USA) reader, with λ_exc_ = 560 nm and λ_em_ = 620 nm. In the case of adherent cultures, 5000 cells/well were seeded in 96-well plates. After 24 h incubation at 37 °C and 5% CO_2_, the medium was removed, and a new medium with 2% FBS containing the various 2-fold dilution of the peptides was added. After a further 30 min, 15 µL of Cell Titer Blue dye was added to each well, and the measurement was performed as above. Viability was calculated relative to a cell with only 2% FBS in the corresponding medium (100% viability). All assays were conducted in triplicate.

### 3.5. Serum Stability

Briefly, 0.5 mL each of human serum (Sigma-Aldrich, St. Louis, MO, USA) and peptide (1 mM in water) were mixed and incubated for 24 h at 37 °C with stirring. Aliquots (0.1 mL) were taken at 0, 1, 5, 10, 30, 120, 360, and 1440 min and treated with 20 µL of trichloroacetic acid (15% (*v*/*v*) in water). The suspension was centrifuged for 30 min at 4 °C and 13,000 rpm, and the supernatant was analyzed by HPLC.

### 3.6. Tolerance in Mice

To determine the maximum lethal dose and appropriate doses for the main study, a sighting study (Figure 2A) was first conducted. Peptides (Ctn[15-34], Ctn[15-34] *re* and Ctn *re*) were administered intraperitoneally (i.p.) at initial doses of 30 mg/kg for Ctn[15-34] and Ctn[15-34] *re* and 3 mg/kg for Ctn *re*. If no toxicity signs were observed, the dose was increased. If toxicity signs or death occurred, a lower dose was administered to another animal until an adequate dose was found. After the sighting study was completed, two main studies were performed.

*Assay 1:* Four randomly distributed groups of five female mice each were used for Ctn[15-34] (30 mg/kg) and Ctn *re* (3 mg/kg) and negative control (HBS). Ctn[15-34] *re* was not included due to spontaneous mortality in the sighting study, even at low concentrations. The peptide was administered i.p., and mice were monitored twice on day 0, once a day until day 4, and then three times a week. The study lasted for 14 days, after which the animals were euthanized, and a systematic necropsy was performed.

*Assay 2*: Two randomly distributed groups of six female mice each were given two different concentrations of Ctn[15-34] (30 and 20 mg/kg). The peptide was sequentially administered i.p., as shown in Figure 3A. Each animal was treated with each dose three times a day for three days. The study was completed when an optimal dose was found that did not induce toxicity or result in very low or reversible toxicity.

### 3.7. Mouse Systemic Infection

A systemic infection model was established in mice, as described before [43]. Briefly, an aliquot from *A. baumannii* stock in 15% glycerol at −80 °C was taken to culture in 3 mL of LB overnight at 37 °C and 250 rpm. After an OD600 reading to adjust bacterial concentration, the culture was diluted with an identical volume of 10% porcine mucin (an enhancer of bacterial infectivity [46]) to a final 1 × 10^8^ CFU/mL concentration. A 10 mL/kg inoculation volume was used for each animal.

### 3.8. Efficacy in Mouse Infection Model

*Assay 1*: A total of 24 mice (12 male, 12 female) randomly distributed into four groups were infected i.p. as described above, then each group was treated with Ctn[15-34] (30 mg/kg), Ctn *re* (3 mg/kg), colistin (10 mg/kg, positive control) or vehicle (HBS, negative control). Treatment (performed blindly) was applied 2 h after infection in a single i.p. dose. All animals were checked for clinical signs twice a day for three days, as described in Table S1. On day 2, animals were euthanized with a 200 mg/kg i.p. dose of pentobarbital.

*Assay 2*: In total, 24 mice (12 male, 12 female) randomly distributed into six groups were infected i.p. as described above, then treated in duplicate groups with Ctn[15-34] (20 mg/kg), colistin (5 mg/kg, positive control) or vehicle (HBS, negative control). Treatment (performed blindly) was applied i.p. 2 h after infection and then every 4 h during day 0, and thrice daily for the next 2 days. All animals were checked for clinical signs twice a day for three days, as described in Table S1. On day 2, animals were euthanized with a 200 mg/kg i.p. dose of pentobarbital.

### 3.9. Evaluation of Body Weight and Clinical Symptoms

Body weight (BW, g) gain (%) was determined at different stages. In the tolerance assays, BW gain was recorded before inoculation and twice a week for 2 weeks. In the efficacy studies, BW gain was likewise measured before inoculation and then twice a day.

Nine clinical parameters were assessed by a scoring system (see Table S1). For each parameter a score from 0 to 3 in increasing severity was used. In the sighting (tolerance) study, the clinical score was measured at 10 min, 30 min, 1 h, 2 h, 4 h, 7 h, 24 h, and 48 h after peptide administration. In assays 1 and 2 of the tolerance study, scoring was recorded twice a week for 2 weeks. In the efficacy study, the clinical score was recorded every 2–3 h on day 0 and twice a day on days 1 and 2. An animal was euthanized if it scored 3 on any parameter, or if a combined score of 7 or above was reached.

### 3.10. Evaluation of Colony-Forming Units (CFU) in Mouse Tissues

CFUs in the spleen and lungs of animals in the efficacy studies were counted after euthanasia. One lung and half of the spleen were extracted from each animal. Animals found dead overnight were excluded. The organs were weighed and homogenized in 1 mL of HBS. Subsequently, six serial 10-fold dilutions were prepared, each dilution seeded on a Petri dish with LB-agar and colonies counted after 16 h incubation at 37 °C. Final CFU counts are expressed relative to organ weight (CFU/g).

### 3.11. Quantification of TNF-α in Mouse Sera by ELISA

In assay 2 of the efficacy study, blood was collected from each animal before euthanasia to assess TNF-α levels. The blood was centrifuged in heparinized tubes at 3200 rpm for 30 min, and the supernatant was collected. A mouse TNF-α ELISA kit was used, following the manufacturer’s instructions.

### 3.12. Stimulation of Human Mononuclear Cells (MNC) by LPS

The MNC stimulation assay by LPS R60 HL185 was based on a previous study [47]. MNC were isolated from heparinized blood samples from healthy donors as described [48]. The cells were resuspended in 1640 RPMI medium and adjusted to a concentration of 5 × 10^6^ cells/mL. Next, 200 μL (1 × 10^6^) of MNC suspension was plated in each of the 96-well plates and then stimulated by incubation with either LPS alone or with a specified peptide/LPS ratio for 4.5 h at 37 °C with 5% CO_2_. Samples were collected and centrifuged, and the supernatant was analyzed for human TNF-α using an ELISA kit.

### 3.13. Histopathology, Tissue Gram Stain and Hematoxylin and Eosin Stain

Following termination of the animals, the following organs were harvested and preserved: right lung, digestive system, pancreas, kidneys, liver, spleen, and brain. These organs were fixed in paraformaldehyde at 4 °C for 24 h.

The tissue processing proceeded through a stepwise procedure. Firstly, the tissues underwent a rinse with formalin for 1.5 h, followed by cleaning with distilled water for 5 min. Subsequently, they were dehydrated using a series of graded ethanol solutions: 80% ethanol for 1 h, 96% ethanol for two consecutive immersions lasting 1 h each, and finally, 100% ethanol for 2 consecutive immersions of 1 h each and an additional immersion for 1.5 h. Following dehydration, the tissues were submerged in xylene for two immersions, each lasting 1.5 h, before being placed in hot liquid paraffin for two consecutive immersions of 1 h each and an additional immersion of 2 h. The treated specimens were embedded in tissue cassettes using a TissueTek embedding console and allowed to cool until the paraffin solidified [49,50].

From each paraffin block, 4 mm sections were cut using a microtome (Leica) and mounted on slides. These slides underwent rehydration through a series of graded alcohols: 100% ethanol for 5 min, 85% ethanol for 5 min, 70% ethanol for 5 min, and distilled water. Following rehydration, a modified Gram stain was performed on the tissue, following the protocol described in [51] (Basic fuchsin, Thermo Fischer; crystal violet solution, Lugol’s iodine solution, Merck). After drying, the sections were analyzed using a microscope (Leica DMR, Leica Biosystems, IL, United States).

Additionally, the samples were stained with hematoxylin at 10% in absolute ethanol (hematoxylin cryst., Sigma Aldrich) and eosin at 0.5% in distilled water (Eosin Y, Sigma Aldrich). Briefly, the samples were hydrated in H_2_O for 10 min and then stained for 5 min in hematoxylin. After two washes in H_2_O, the samples were introduced in 70% ethanol with 1% of hydrochloric acid for 30 s. The samples were washed again and stained with eosin for 5 min. Finally, the samples were dehydrated and mounted on gelatinized glass slides. Samples were visualized using a light microscope (Olympus BX51, Tokyo, Japan).

### 3.14. Statistical Analysis

ANOVA tests were performed for statistical comparison between groups. The ELISA linear curve was fitted by linear regression (Figures S1 and S2).

## 4. Conclusions

The goal of this study was to assess to what extent the promising in vitro prospects of peptides Ctn *re*, Ctn[15-34] and Ctn[15-34] *re* [38] could be actualized in vivo as a further step toward therapeutic application. The results obtained in this work are a sobering reminder that the gap between in vivo and in vitro studies can be, at times, frustratingly broad. We have encountered low efficacy for Ctn *re* and Ctn[15-34] and an inflammatory response leading to spontaneous death for Ctn[15-34] *re*. These adverse outcomes, exceeding even low-key expectations from our side, preclude for the moment any further developments on these peptides.

The in vivo failure of Ctn[15-34] as an AMP might be likely related to interactions with serum proteins such as AFM, ApoL1, ApoM, DBP, and CBG via its C-terminal hydrophobic tail [36]. While beneficial in shielding the peptide from protease degradation, too extensive binding to serum proteins might limit the amount of peptide available for interaction with bacteria. Exploring the sensitive balance between peptide and serum proteins, and its impact on antibacterial action, is a must for future AMP studies.

## Figures and Tables

**Table 1:** Sequence and properties of peptides in this study.

Peptide	Sequence *	MIC (μM) *A. baumannii*	IC_50_ (μM)	t_1/2_ (min)
Ctn	KRFKKFFKKVKKSVKKRLKKIFKKPMVIGVTIPF	0.78	14.32	71
Ctn *re*	fpitvGivmpkkfikklrkkvskkvkkffkkfrk	1.56	4.3	211
Ctn[15-34]	KKRLKKIFKKPMVIGVTIPF	0.78	>100	770
Ctn[15-34] *re*	fpitvGivmpkkfikklrkk	0.39	43	921

* L- and D-residues in uppercase and lowercase, respectively; all peptides in C-terminal amide form.

## Data Availability

Data are contained within the article.

## Supplementary Materials

The following supporting information can be downloaded at: https://www.mdpi.com/article/10.3390/ijms25189773/s1.
