# Supplementary material for: From In Vitro Promise to In Vivo Reality: An Instructive Account of Infection Model Evaluation of Antimicrobial Peptides
Source: Int J Mol Sci. 2024 Sep 10;25(18):9773. doi: 10.3390/ijms25189773 (PMC11431785; doi:10.3390/ijms25189773)

Supplementary information

In Vivo **Evaluation of Snake Venom-Derived Antimicrobial Peptides in an** ***Acinetobacter baumannii*** **Infection Model**

1. *In vivo* data

Table S1. Assessment of general health status.

Clinical assessment	Score definition*
Body weight	0: body weight gain<br>1: no body weight gain/body weight loss <10% of the initial body weight<br>2: body weight loss between 10-20%<br>3: body weight loss >20%
Motor activity	0: normal<br>1: decreased motor activity, lameness, ataxia<br>2: need to be forced to stand up, partial paralysis<br>3: prostration, complete paralysis, dose no stand up when forced
General appearance	0: normal<br>1: less grooming than normal, mild piloerection<br>2: moderate piloerection and lesions in the coat, hunched posture<br>3: severe piloerection and lesions in the coat (ulcerative dermatitis)
Behaviour	0: normal/social behaviour<br>1: excitation/depressed<br>2: exacerbated reactions to external stimuli/no reaction to external stimuli<br>3: aggressiveness/stupor
Secretions	0: no secretion<br>1: mild secretion<br>2: severe secretion<br>3: haemorrhage/purulent secretion
Hydration status	0: normal<br>1: long skin-tent duration<br>3: long skin-tent duration + enophthalmos
Breathing	0: normal<br>1: mild dyspnoea or tachypnoea<br>3: moderate dyspnoea or tachypnoea + prostration
Hypovolemia signs	0: mucous rose-coloured<br>1: mucous paleness<br>2: moderate mucous paleness and dry<br>3: moderate mucous paleness and dry + tachypnoea
Urination and defecation	1: faeces and/or urine in very small quantities or slightly dark/orange or another colour appearance<br>2: faeces and/or dark/pink urine excretion indication blood. appreciation of dilated bladder and/or hardened abdomen<br>3: dark faeces and dark pink urine excretion indication blood. appreciation of dilated bladder and hardened abdomen, stained perineal area. completely liquid faeces

* Sum of the score: 0-2: No alterations. Procedure will be continued; 3-6: Monitoring will be intensified. ≥7: Euthanasia will be considered.

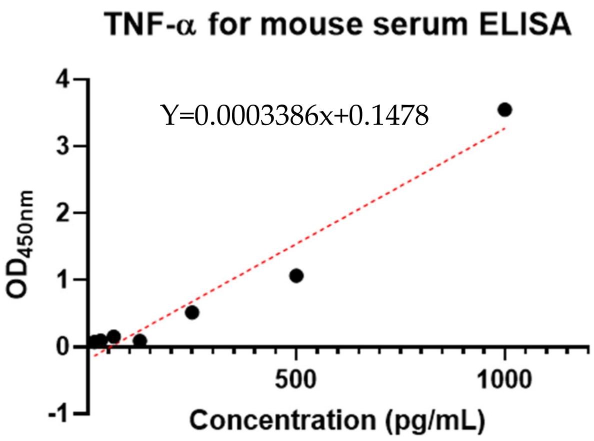

Figure S1. Standard curve of TNF-α for mouse serum to test by ELISA. The curve and the equation of the TNF-α concentration relative to OD_{450} were obtained from the curve fit by linear regression. (R^2=0.9542)

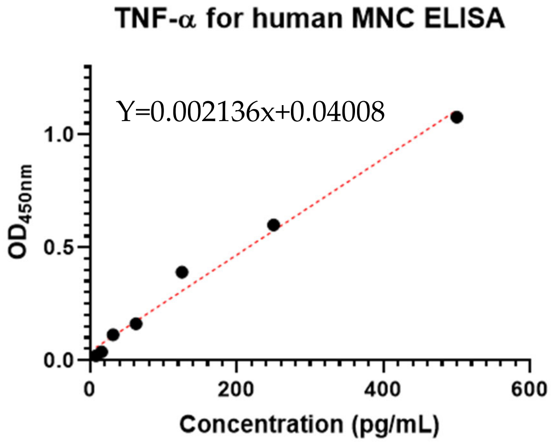

Figure S2. Standard curve of TNF-α for human MNC to test by ELISA. The curve and the equation of the TNF-α concentration relative to OD_{450} were obtained from the curve fit by linear regression. (R^2=0.9871)

2. Analytical data

Table S2. Peptide analytical data.

Peptide[1]	Theoretical mass (Da)	Experimental mass (Da)[2]	HPLC retention time (min)[3]	Purity[4]
Ctn	4151.36	4151.40	7.0	97%
Ctn *re*	4151.36	4151.00	8.1	97%
Ctn [15-34]	2371.08	2370.40	6.5	99%
Ctn [15-34] *re*	2371.08	2370.60	6.6	97%

[1] All peptides are C-terminal carboxamide; [2] Determined by LC-MS; [3] Elution was carried out with a 10 to 50 linear gradient over 15 min; [4]Determined by analytical HPLC.

HPLC-MS analysis. Left: HPLC traces of purified peptides. Right: ESI-MS spectra of purified peptides.

Ctn: KRFKKFFKKVKKSVKKRLKKIFKKPMVIGVTIPF

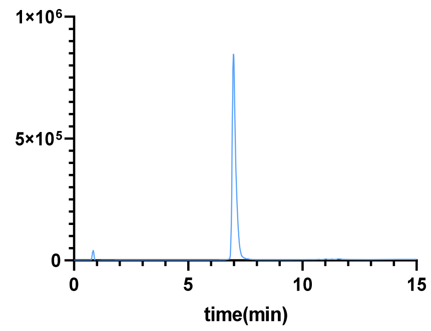

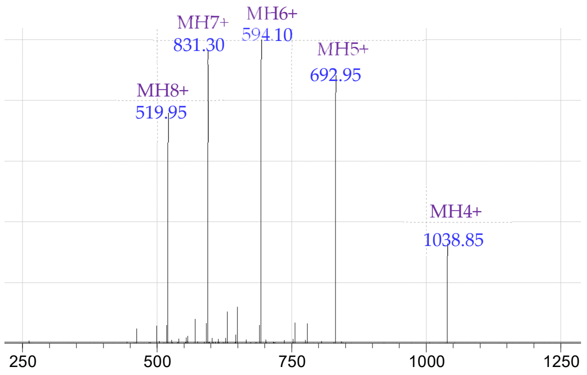

Ctn *re*: fpitvGivmpkkfikklrkkvskkvkkffkkfrk

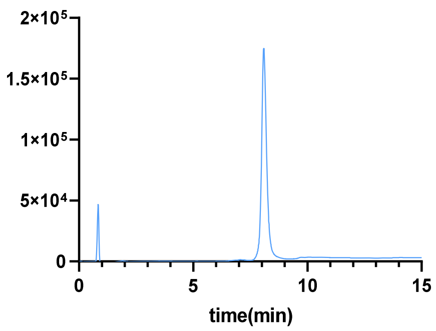

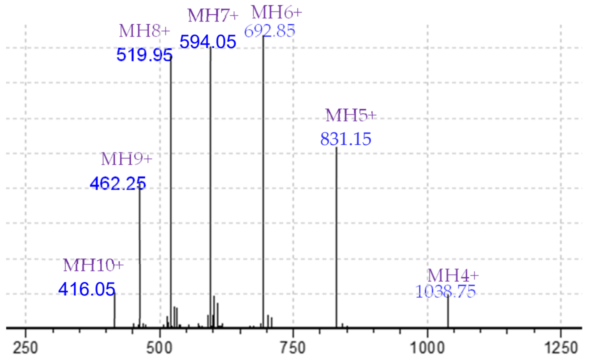

Ctn[15-34]: KKRLKKIFKKPMVIGVTIPF

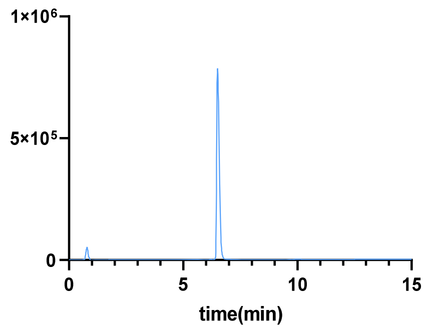

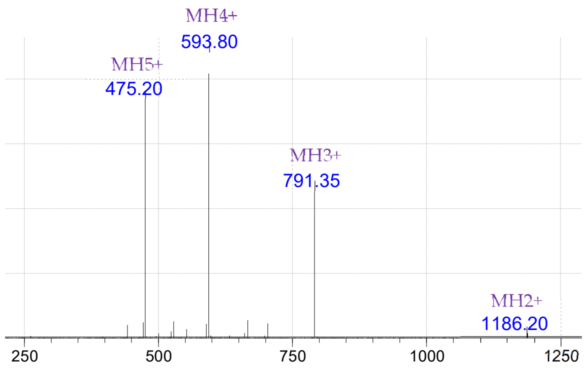

Ctn[15-34] *re*: fpitvGivmpkkfikklrkk

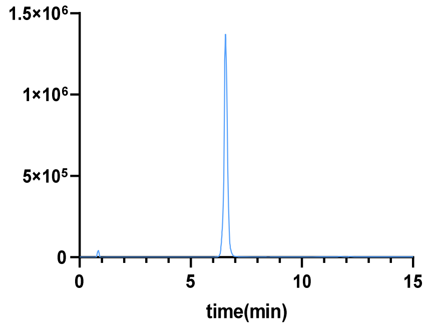

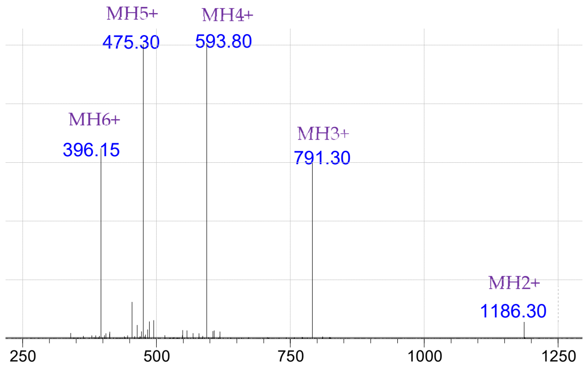

Supplement: Supplementary file 1 [file ijms-25-09773-s001.zip › ijms-3164621-supplementary.pdf]
